# Complete chloroplast genome of green tide algae *Ulva flexuosa* (Ulvophyceae, Chlorophyta) with comparative analysis

**DOI:** 10.1371/journal.pone.0184196

**Published:** 2017-09-01

**Authors:** Chuner Cai, Lingke Wang, Lingjie Zhou, Peimin He, Binghua Jiao

**Affiliations:** 1 College of Marine Ecology and Environment, Shanghai Ocean University, Shanghai, China; 2 National Demonstration Center for Experimental Fisheries Science Education (Shanghai Ocean University), Shanghai, China; 3 Marine Biomedicine Institute, Second Military Medical University, Shanghai, China; 4 Department of Marine Sciences, University of Connecticut, Groton, Connecticut, United States of America; Chinese Academy of Medical Sciences and Peking Union Medical College, CHINA

## Abstract

*Ulva flexuosa*, one kind of green tide algae, has outbroken in the Yellow Sea of China during the past ten years. In the present study, we sequenced the chloroplast genome of *U*. *flexuosa* followed by annotation and comparative analysis. It indicated that the chloroplast genomes had high conservation among *Ulva* spp., and high rearrangement outside them. Though *U*. *flexuosa* was closer to *U*. *linza* than *U*. *fasciata* in phylogenetic tree, the average Ka/Ks between *U*. *flexuosa* and *U*. *linza* assessed by 67 protein-coding genes was higher than those between *U*. *flexuosa* and other species in *Ulva* spp., due to the variation of *psb*Z, *psb*M and *ycf*20. Our results laid the foundation for the future studies on the evolution of chloroplast genomes of *Ulva*, as well as the molecular identification of *U*. *flexuosa* varieties.

## Introduction

The macroalgae representing the cosmopolitan green algae *Ulva flexuosa* Wulfen species have been found in Europe since the mid-1800s [1], which are very common and regularly encountered in fresh waters, slightly saline waters, and estuaries [2], containing the characteristics of transitional species. As one of the original species in green tide outbreak in Yellow Sea of China since 2008, *U*. *flexuosa* has attracted global attention for its fast growth if given appropriate nutrient supply, with maximum growth rate of 73% d^-1^ at room temperature under light intensity of 70 μmol·m^-2^·s ^-1^ [3]. On the other hand, culturists in Brazilian are interested in the sulfated polysaccharide ulvan which triggers plant defenses against disease and can be obtained from *U*. *flexuosa* within a production cycle of 15 days [4].

*U*. *flexuosa* belongs to Ulvophyceae, with thalli composed of uninucleate cells, and is difficult to be set apart from species among *Ulva* spp., since the latter is often sheet like in external morphology [5]. Furthermore, the vegetative cells arrangement of the mature thalli show tendency towards distortions determined by the calcium carbonate crystals which increase the difficulty for identification [6, 7]. Then, the rRNA ITS region, 5S and partial *rbc*L gene sequences are applied to demonstrate sequence variation within species or even subspecies [2, 8]. The chloroplast genome sequencing may provide more molecular markers.

In this study, we present the first complete chloroplast genome of *U*. *flexuosa*. The genome was sequenced, assembled, and annotated as circular-mapping DNA molecules. Additionally, we analyzed the repeat sequence using MISA, Tandem Repeats Finder, and REPuter. Furthermore, *U*. *flexuosa* chloroplast genome was compared to others using phylogenomic investigation, Mauve and blastn program provided by EMBOSS to assess the monophyly of the Ulvophyceae and relationships of ulvophytes with other core chlorophyta clades.

## Materials and methods

### Sampling and species identification

Samples of green tide algae were collected from the southern Yellow Sea near Rudong sea area, Jiangsu (N32°31′04.10″ E121°25′11.99″), where lavers breed every year and no endangered or protected species is involved. So no specific permissions were required for these locations and activities. After repeated washing with seawater, the samples were dried in the shade to the moisture content of 30–40%. Then, they were transported to the laboratory in an insulated specimen box at 4°C.

Healthy individual algae were selected to be washed several times with sterile seawater autoclaved at 121°C for 15 minutes. After removing surface attachments, they were cultured in VSE medium [9] for 2d at 24°C under a 12:12 h LD photoperiod and photon flux 130–160 μmolm^-2^ s^-1^.

After being put into the white porcelain tray with a small amount of seawater, algae were gently expanded with a brush to exhibit the main external form. Slices, made through freehand transverse section, were placed under an Olympus optical microscope for observation.

Algae matching the description of *U*. *flexuosa* were used to extract DNA using the DNAsecure Plant Kit (Tiangen Biotech, Beijing) [10] and subjected to PCR amplification using primers specific for ITS [11] and 5S rDNA [12]. The amplified sequences were sent to Shanghai Biology Engineering Technology Service Co., Ltd. for shotgun sequencing.

### DNA sequencing and assembly

After comparison, the chloroplast genome of *Ulva* sp. UNA00071828 [13] was used as a model for primer sequence design (S1 Table). After preliminary experiment, the primers were re-integrated. Then the whole genome was divided into 15 segments (S2 Table) for PCR amplification under the following conditions: predenaturation at 94°C for 1 min, 35 cycles of amplification at 94°C for 30 s, 55°C for 30 s and 68°C for 8 min, followed by a final extension at 68°C for 15 min. The PCR reaction mixture contained 0.5μl of DNA polymerase (5U/μl), 5μl of Buffer (Mg^2+^ Plus) (10×), 8μl of dNTP Mixture (2.5mM each), 0.2–1.0μM (final concentration) of forward primer, 0.2–1.0μM (final concentration) of reverse primer, and purified chloroplast DNA (<1 μ g). RNase-free water was added to increase the final reaction volume to 50 μl. Wherein Takara *LA Taq* polymerase was used for fragments longer than 4,000 bp, Takara *Ex Taq* was used for the others. The PCR products of long segments were broken with ultrasound, connected to vector PMD19-T, and transformed into *E*. *coli* DH5a. Positive clones were selected for shotgun sequencing by ABI3730 sequencer. The correctness of the assembly was validated further by mapping all raw sequence reads to the assembly using phred V1.09 program [14].

### Species verification of *U*. *flexuosa*

In order to verify species *U*. *flexuosa* by comparison of *rbc*L and *tuf*A genes, Chloroplast *rbc*L and *tuf*A genes in *Ulva* spp. were extracted from genebank (S3 Table) followed by blastn with corresponding *U*. *flexuosa* genes using DNAMAN. Poorly aligned positions were removed using the Gblocks server [15, 16] with the least stringent settings: allowing smaller final blocks, gap positions within the final blocks, less strict flanking positions and many contiguous non-conserved positions. Gblocks removed 87 of 1430 positions for *rbc*L and 458 of 1231 positions for *tuf*A, 1343pb and 773bp alignments were remained respectively for phylogenetic analysis. A UPGMA tree was made by MEGA 6.06 [17] for each alignment with number of differences genetic distance model and 1000 bootstrap replicates [18]. *Blidingia minima* (AF387109) and *Monostroma grevillei* (GU183089) were used as outgroups for *rbc*L, while *B*. *minima* (HQ610329) and *Monostroma* sp. (HQ610262) were used for *tuf*A. Additionally, the pairwise distances (bp) were calculated to aid in the identification of this *Ulva* species.

### Genome annotation and codon usage analysis

The CpGAVAS web service was used to annotate the *U*. *flexuosa* chloroplast genome [19]. Cutoffs for the E-values of BLASTN and BLASTX were 1e^-10^. The number of top hits included in the reference gene sets for annotation after the pre-filtering step was 10. Meanwhile, tRNA genes were identified using tRNAscan-SE [20] and ARAGORN [21]. Manual corrections on the positions of the start and stop codons, and for the intron/exon boundaries were aided by blastn with other chloroplast genomes in Ulvophyceae. The inverted repeat (IR) sequences were predicted by Gepard-1.40 analysis [22]. Moreover, the circular chloroplast genome map of *U*. *flexuosa* was drawn using Organellar Genome DRAW [23]. Furthermore, codon usage and GC content were analyzed using the Cusp and Compseq programs provided by EMBOSS [24]. Final genome assembly and genome annotation results were deposited in GenBank (accession number: KX579943).

### Repeat sequence analysis

SSRs were detected using MISA Perl Script available at “http://pgrc.ipk-gatersleben.de/misa/”, with the following thresholds: eight repeat units for mononucleotide SSRs, four repeat units for di- and trinucleotide repeat SSRs, and three repeat units for tetra-, penta-, and hexa-nucleotide repeat SSRs. Tandem repeats were analyzed using Tandem Repeats Finder [25] with parameter settings of two for matches and seven for mismatches and indels. The minimum alignment score and maximum period size were set at 50 and 500, respectively. All the identified repeats were manually verified and nested or redundant results were removed. REPuter [26] was employed to identify the IRs in *U*. *flexuosa* by forward, reverse, complementary and palindromic alignment. The minimal repeat size was set at 30 bp, and the cutoff for similarities among the repeat units was set at 100%.

### Phylogenomic analysis

A total of 67 complete chloroplast DNA sequences belonging to algae were obtained from Genbank. For the phylogenetic analysis, 24 nucleotide sequences shared among all these 67 species and *U*. *flexuosa* were aligned by Clustal-Omega with default parameters. The 24 genes are *atp*A, *atp*B, *atp*E, *atp*F, *pet*B, *pet*D, *pet*G, *psa*B, *psb*B, *psb*D, *psb*E, *psb*F, *psb*H, *psb*J, *psb*K, *psb*L, *psb*N, *rbc*L, *rpl*2, *rpl*20, *rpl*36, *rpo*A, *rps*8 and *ycf*3. The appropriate model of evolution for each gene determined by jModelTest 2.1 was GTR+I+G, which was reassessed by PAUP 4.0a152 (P value = 1 − (99/100) = 0.010000). Then, the evolutionary history was inferred using the Maximum Likelihood method implemented in RaxML. The detailed parameters were “raxmlHPC-PTHREADS -f a -x 12345 -# 1000 -m GTRGAMMA -s dna.phy -n nex -p 12345 -q part.txt -T 6”. The tree with the final ML Optimization Likelihood (-548221.173374) was shown. The significance level for the phylogenetic tree was assessed by bootstrap testing with 1000 replications. Finally, MrBayes were used to calculate Bayesian posterior probabilities for another line of statistical support, in which the main parameters are: ngen = 2000000, samplefreq = 100, nchains = 4, nst = 6, rates = gamma. The results were published to TreeBASE <treebase.org> (submission number: 21293).

### Rearrangements in genomes

Genome rearrangement was aligned with progressive Mauve by Mauve 2.4.0 [27]. 10 species were applied including *U*. *flexuosa* (KX579943), *U*. *linza* (NC_030312), *U*. *fasciata* (NC_029040), *Ulva* sp. UNA00071828 (KP720616), *Pseudendoclonium akinetum* (NC_008114), *Gloeotilopsis sterilis* (NC_025538), *Tydemania expeditionis* (NC_026796), *Bryopsis plumosa* (NC_026795), *Bryopsis hypnoides* (NC_013359) and *Ostreobium* sp. (KU979013) [28, 29].

### Comparative genome analysis

Genome synteny was analyzed between the chloroplast genomes of *U*. *flexuosa* (KX579943) and those of *B*. *hypnoides* (NC_013359), *B*. *plumosa* (NC_026795), *Gloeotilopsis planctonica* (KX306824), *Gloeotilopsis sarcinoidea* (KX306821), *G*. *sterilis* (NC_025538), *Ostreobium* sp. (KU979013), *P*. *akinetum* (NC_008114), *T*. *expeditionis* (NC_026796), *Ulva fasciata* (NC_029040), *Ulva linza* (NC_030312), and *Ulva* sp. UNA00071828 (KP720616) using blastn program provided by EMBOSS. The detailed parameters were “blastn -query genome1.fasta -db genome2.fasta -out result.o -outfmt 7”. The homologous regions were visualized using a web-based genome synteny viewer GSV [30].

## Results

### Species verification of *U*. *flexuosa*

**Fig 1:**
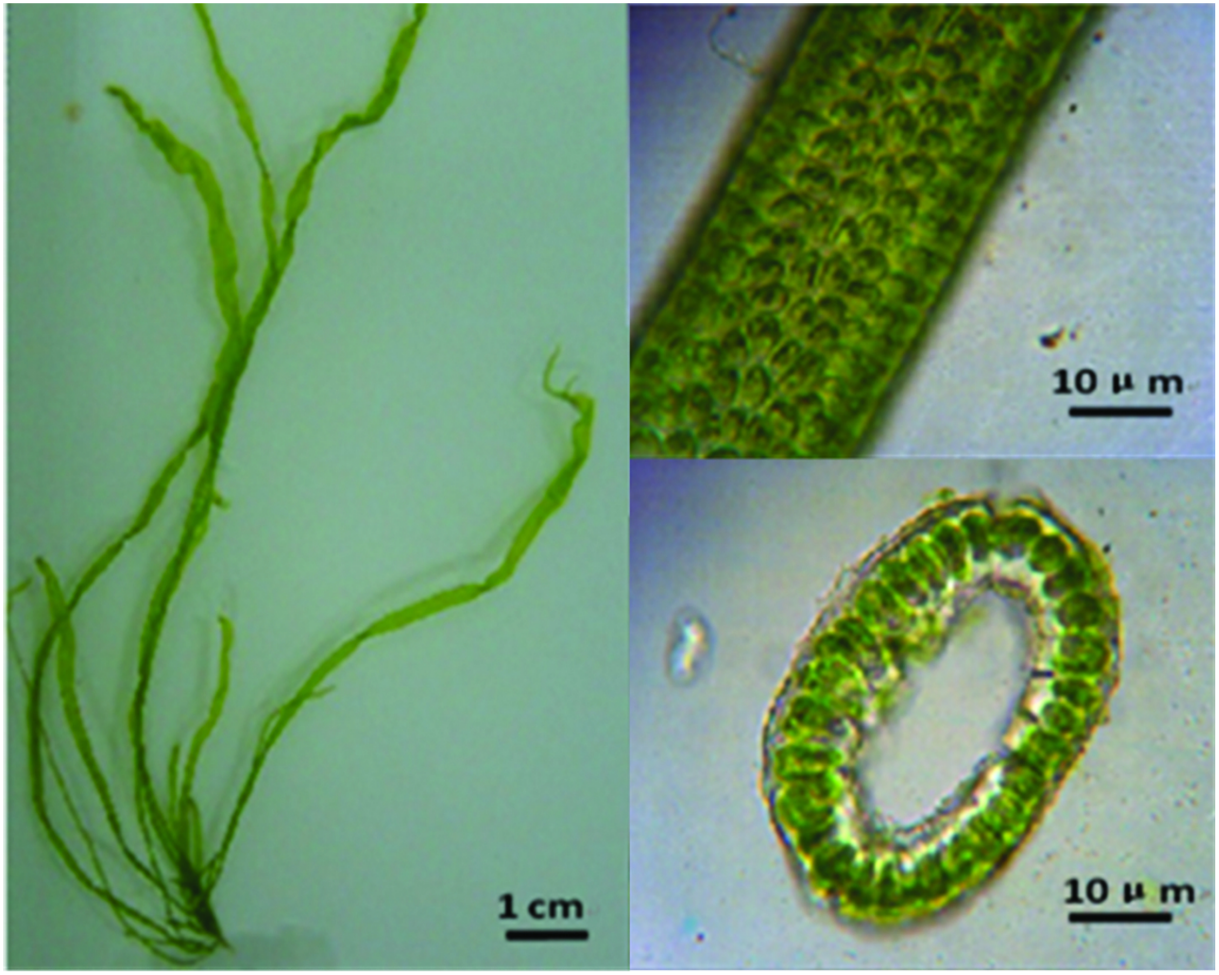
Morphological characteristics of *U*. *flexuosa*.

Four samples of algae were identified as most likely belonging to *U*. *flexuosa*, based on morphology and ITS sequence. The bright green thallus is tubular at the base but flattened above. It grows into long, narrow, ruffled blades two cell layers thick, 0.7–1 cm wide and up to 6–18 cm tall. Cells are 11–15 μm in diameter. Additionally, each cell contains 1–3 pyrenoids, or even 4–5 occasionally (Fig 1). The four samples had highest sequence similarity with *U*. *flexuosa* (AB097646.1, HM031176.1), with 99.7% ITS sequence identity.

**Fig 2:**
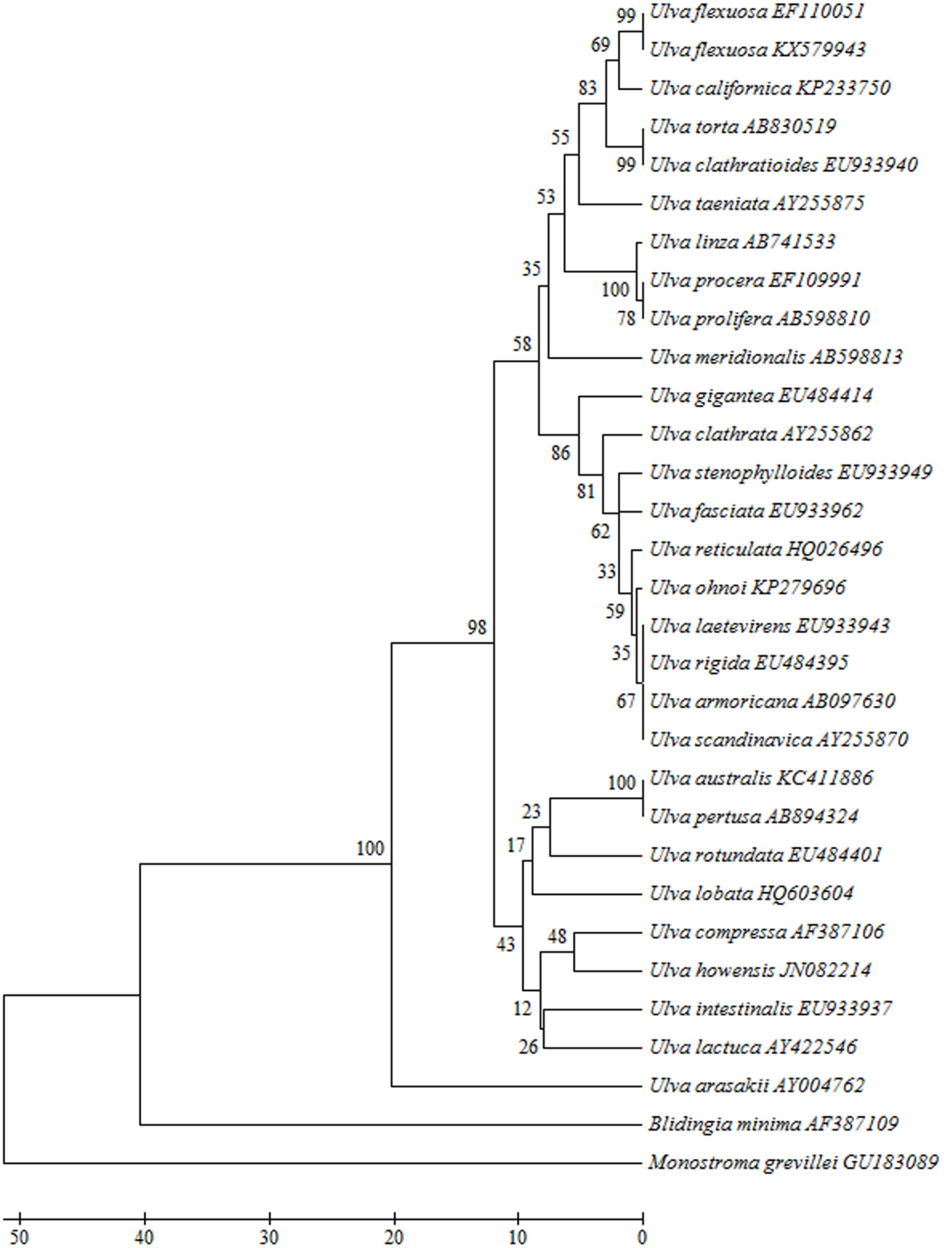
Evolutionary relationships of taxa from *Ulva* based on the *rbc*L gene.

**Fig 3:**
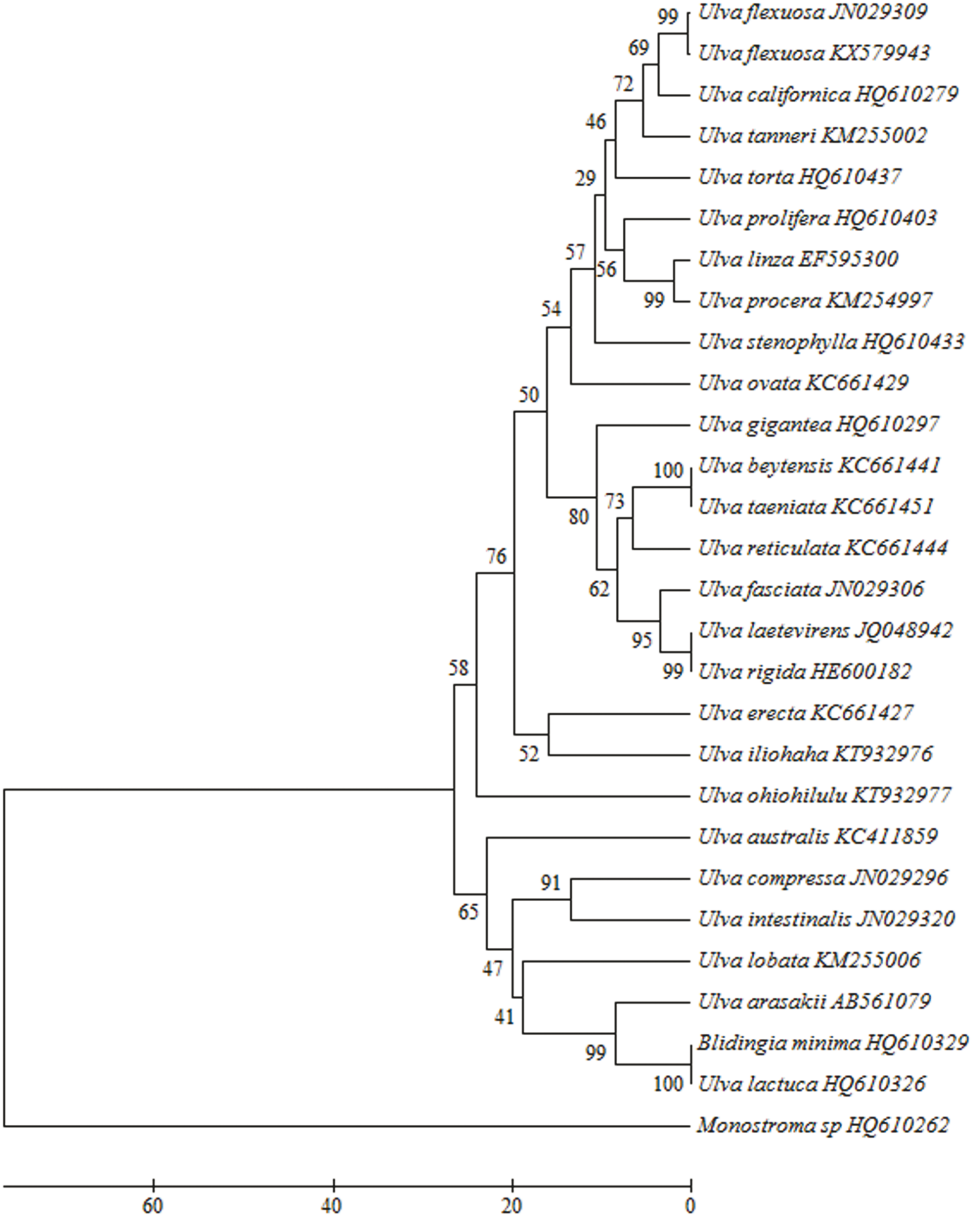
Evolutionary relationships of taxa from *Ulva* based on the *tuf*A gene.

The chloroplast *rbc*L (EF110051) and *tuf*A (JN029309) sequences from genus *Ulva* species in NCBI had highest consistency with *U*. *flexuosa* samples in this report according to UPGMA phylogenetic tree, both with credibility of 99% (Figs 2 and 3). The pairwise distances were zero and one (S1 and S2 Fig). Combined with its morphological characteristics, it could be confirmed as *U*. *flexuosa*.

### General features of the *U*. *flexuosa* chloroplast genome

**Fig 4:**
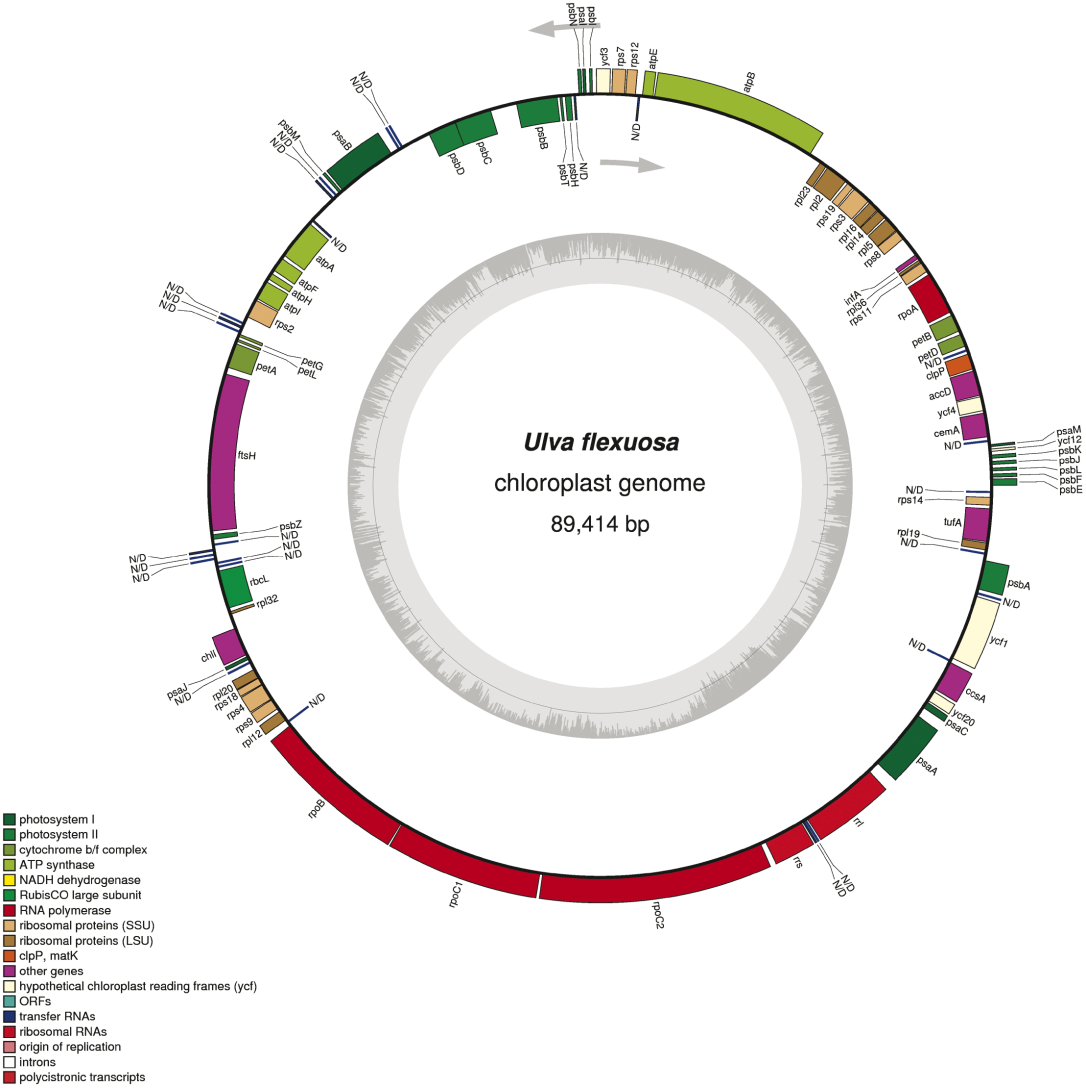
Schematic representation of the *U*. *flexuosa* chloroplast genome using OGDRAW. The predicted genes are shown and colors represent functional classifications, which are shown at the left bottom. The genes drawn outside the circle are transcribed counterclockwise. The inner circle shows the GC content. IR regions are not found according to Gepard-1.40 analysis.

The complete chloroplast genome sequence is 89414 bp long with no IR region. Moreover, a total of 99 genes were identified, including 71 protein coding genes, 26 tRNA genes, and 2 genes for large and small ribosomal RNA (*rrl* and *rrs*). In addition to the conserved genes, two conserved open reading frames were determined (Table 1). The general structure and locations of the 99 genes in the chloroplast genome are depicted in Fig 4. No intron has been predicted. Overall, 72.0% of the *U*. *flexuosa* chloroplast genome sequence is composed of genes that code proteins. The overall GC content of the *U*. *flexuosa* chloroplast genome is 25.0%, whereas the protein-coding regions account for 26.5%. Within the protein-coding regions, the GC contents for the first, second and third positions of the codons occupy 35.62%, 31.34% and 12.43%, respectively.

**Table 1:** Genes predicted in the chloroplast genome of *U*. *flexuosa*.

Category of genes	Group of genes	Name of genes
**Self-replication**	tRNA genes	26 *trn* genes
	Small subunit of ribosome	*rps*11, *rps*12, *rps*14, *rps*18, *rps*19, *rps*2, *rps*3, *rps*4, *rps*7, *rps*8, *rps*9
	Large subunit of ribosome	*rpl*12, *rpl*14, *rpl*16, *rpl*19, *rpl*2, *rpl*20, *rpl*23, *rpl*32, *rpl*36, *rpl*5
	DNA dependent RNA polymerase	*rpo*A, *rpo*B, *rpo*C1, *rpo*C2
	rRNA genes	*rrl*(23S), *rrs*(16S)
**Genes for photosynthesis**	Subunits of ATP synthase	*atp*A, *atp*B, *atp*E, *atp*F, *atp*H, *atp*I
	Subunits of cytochrome b/f complex	*pet*A, *pet*B, *pet*D, *pet*G, *pet*L
	Subunits of photosystem I	*psa*A, *psa*B, *psa*C, *psa*I, *psa*J, *psa*M, *ycf*3, *ycf*4
	Subunits of photosystem II	*psb*A, *psb*B, *psb*C, *psb*D, *psb*E, *psb*F, *psb*H, *psb*I, *psb*J, *psb*K, *psb*L, *psb*M, *psb*N, *psb*T, *psb*Z, *ycf*12
	Subunit of rubisco	*rbc*L
	Subunit of protochlorophyllide reductase	*chl*I
**Other genes**	Subunits of Acetyl-CoA-carboxylase	*acc*D
	C-type cytochrome synthesis gene	*ccs*A
	Envelope membrane protein	*cem*A
	Protease	*clp*P
	Elongation factor	*tuf*A
	Zinc metalloprotease	*fts*H
	Translation initiation factor	*inf*A
**Genes of unknown function**	Conserved open reading frames	*ycf*1, *ycf*20

The codon usage and codon-anticodon recognition pattern of the *U*. *flexuosa* chloroplast genome are summarized in S4 Table. The 29 tRNA genes include codons corresponding to all 20 amino acids that are necessary for biosynthesis. UUA (encoding Leu) is the most frequently used codon, followed by AAA (Lys), AAU (Asn), AUU (Ile), UUU (Phe), GAA (Glu), UAU (Tyr), GGU (Gly) and GAU (Asp). These nine codons account for 50% of all codons, and the third base pair for all of these is either A or U. In addition, optimal codons for all amino acids end either in A or U, with the exception of Met and Trp, both of which have a single codon (AUG and UGG). All the results are consistent with the high AT content of *U*. *flexuosa* chloroplast DNA (75.0%).

### Repeat and SSR analysis

SSRs are valuable molecular markers of high-degree variations within the same species and have been used in population genetics and polymorphism investigation [31]. We analyzed the occurrence, type, and distribution of SSRs in the *U*. *flexuosa* chloroplast genome and the distribution of SSRs in 11 other chloroplast genomes belonging to Ulvophyceae. In total, 236 SSRs were identified in the *U*. *flexuosa* chloroplast genome (S5 Table and Table 2). Among these SSRs, the majority consisted of mono- and di- nucleotide repeats, which were found 167 and 40 times, respectively. Tri- (19), tetra- (9), hexa- nucleotide repeat sequences (1) were found with lower frequency. This observed pattern is similar to those observed in 3 chloroplast genomes of other species belonging to Ulvaceae (Table 3). All the mononucleotide repeat sequences consisted of A/T repeats. Similarly, most dinucleotide repeat sequences consisted of AT/AT repeats (92.5%) (S5 Table). Our findings are in agreement with the previous findings that the chloroplast SSRs are generally composed of short polyA or polyT repeats and rarely contain tandem G or C repeats [32]. In this study, we also analyzed the locations of 29 tri-, tetra- and hexa- nucleotides in the chloroplast genome, and the results are shown in Table 2. Among these nucleotides, 15 are localized in the intergenic regions, 13 are in the coding regions, and the last one is stretching across intergenic regions and coding regions.

**Table 2:** Distribution of tri-, tetra-, penta- and sex- nucleotide SSR loci in the chloroplast genome of *U*. *flexuosa*.

SSR type	SSR sequence	Start	End	Location
**tri**	(TAA)_4_	704	715	IGS^a^(*psbL-psbJ*)
**tri**	(ATA)_4_	28544	28555	CDS^b^(*psbD*)
**tri**	(ATA)_6_	32962	32979	IGS(*trnH-trnS*)
**tri**	(ATA)_4_	36822	36833	IGS(*atpH-atpI*)
**tri**	(ATT)_4_	44068	44079	CDS(*ftsH*)
**tri**	(TAA)_4_	47408	47419	CDS(*trnE*),IGS(*trnE-trnM*)
**tri**	(TAA)_4_	47423	47434	IGS(*trnE-trnM*)
**tri**	(TAA)_5_	47438	47452	IGS(*trnE-trnM*)
**tri**	(TAA)_4_	47465	47476	IGS(*trnE-trnM*)
**tri**	(ATT)_6_	51077	51094	IGS(*chlI-psaJ*)
**tri**	(AAT)_4_	60077	60088	CDS(*rpoC1*)
**tri**	(TAA)_4_	60436	60447	CDS(*rpoC1*)
**tri**	(TTA)_4_	61640	61651	CDS(*rpoC1*)
**tri**	(ATA)_4_	67042	67053	CDS(*rpoC2*)
**tri**	(ATA)_4_	69097	69108	CDS(*rpoC2*)
**tri**	(ATA)_4_	71404	71415	CDS(*rpoC2*)
**tri**	(TAA)_4_	71651	71662	CDS(*rpoC2*)
**tri**	(CAA)_4_	78378	78389	CDS(*psaA*)
**tri**	(TAA)_4_	89011	89022	IGS(*rps14-trnG*)
**tetra**	(AATT)_3_	9084	9095	IGS(*infA-rps8*)
**tetra**	(CTTT)_3_	25471	25482	IGS(*psbB-psbC*)
**tetra**	(AATT)_3_	29352	29363	IGS(*psbD-trnM*)
**tetra**	(TAAT)_3_	41783	41794	CDS(*ftsH*)
**tetra**	(ATAA)_3_	46377	46388	IGS(*ftsH-psbZ*)
**tetra**	(AAAT)_3_	72259	72270	CDS(*rpoC2*)
**tetra**	(GTAG)_3_	76586	76597	CDS(*rrl*)
**tetra**	(TTTA)_3_	80831	80842	IGS(*psaA-psaC*)
**tetra**	(ATTA)_3_	86695	86706	IGS(*psaA-trnT*)
**sex**	(TAATTT)_3_	10101	10118	IGS(*rps8-rpl5*)

^a^intergenic spacer region.

^*b*^coding sequences.

**Table 3:** Distributions of SSRs in the 11 chloroplast genomes belonging to Ulvophyceae.

Species Name	Accession	Inverted Repeat	Number of SSRs						
			1	2	3	4	5	6	Total
***Bryopsis hypnoides***	NC_013359	No	257	32	1	7	2	1	300
***Bryopsis plumosa***	NC_026795	No	124	18	1	6	1	1	151
***Gloeotilopsis planctonica***	KX306824	No	650	14	6	11	1	3	685
***Gloeotilopsis sarcinoidea***	KX306821	No	806	25	7	14	0	17	869
***Gloeotilopsis sterilis***	NC_025538	Yes	302	18	5	14	1	2	342
***Ostreobium* sp**	KU979013	No	67	20	0	5	0	0	92
***Pseudendoclonium akinetum***	NC_008114	Yes	445	40	8	10	2	1	506
***Tydemania expeditionis***	NC_026796	No	101	26	4	3	0	3	137
***Ulva fasciata***	NC_029040	No	162	34	16	14	3	1	230
***Ulva linza***	NC_030312	No	174	38	19	7	4	5	247
***Ulva* sp**	KP720616	No	166	38	20	18	10-	5	247

Using Tandem Repeats Finder, five repeats were detected in the *U*. *flexuosa* chloroplast genome. All of these repeats were between 10 and 15 bp in length. The intergenic spacers between *rpoC2* and *rrs*, *psbB* and *psbC* possessed two copies of the longest tandem repeats, respectively (15 bp). Most of the repeated sequences were located in the intergenic spacer besides one repeat which was located among coding sequences of *rpoC2* (Table 4).

**Table 4:** Repeat sequences identified in the chloroplast genome of *U*. *flexuosa*.

Repeat Number	Repeat size (bp)	Type	Location	Repeat Unit sequence
**1**	10	T	IGS^a^ (*trnI-psbH*)	(ATTATTTTAT)_3_
**2**	13	T	CDS^b^(*rpoC2*)	(AATTTAAAGAAAA)_2_
**3**	15	T	IGS(*rpoC2-rrs*)	(GTTTTAATATAAATT)_2_
**4**	15	T	IGS(*psbB-psbC*)	(AAATTAAAAAATAT)_2_
**5**	12	T	IGS(*trnV-rpoB*)	(ATTATTAATTAA)_2_
**6**	31	F	IGS(*trnE-trnM*)	TAATAATATTAATAATAATAATATTAATAAT
**7**	30	F	IGS(*rpl32-chlI*)	TTTATTAATATATACTAAAATTATTAATTA

^a^intergenic spacers.

^b^coding sequences.

Two forward repeats were identified using REPuter with a size cutoff of 30 bp, which were between *trnE* and *trnM* (31 bp), *rpl32* and *chlI* (30 bp) (Table 4).

### Phylogenomic analyses

**Fig 5:**
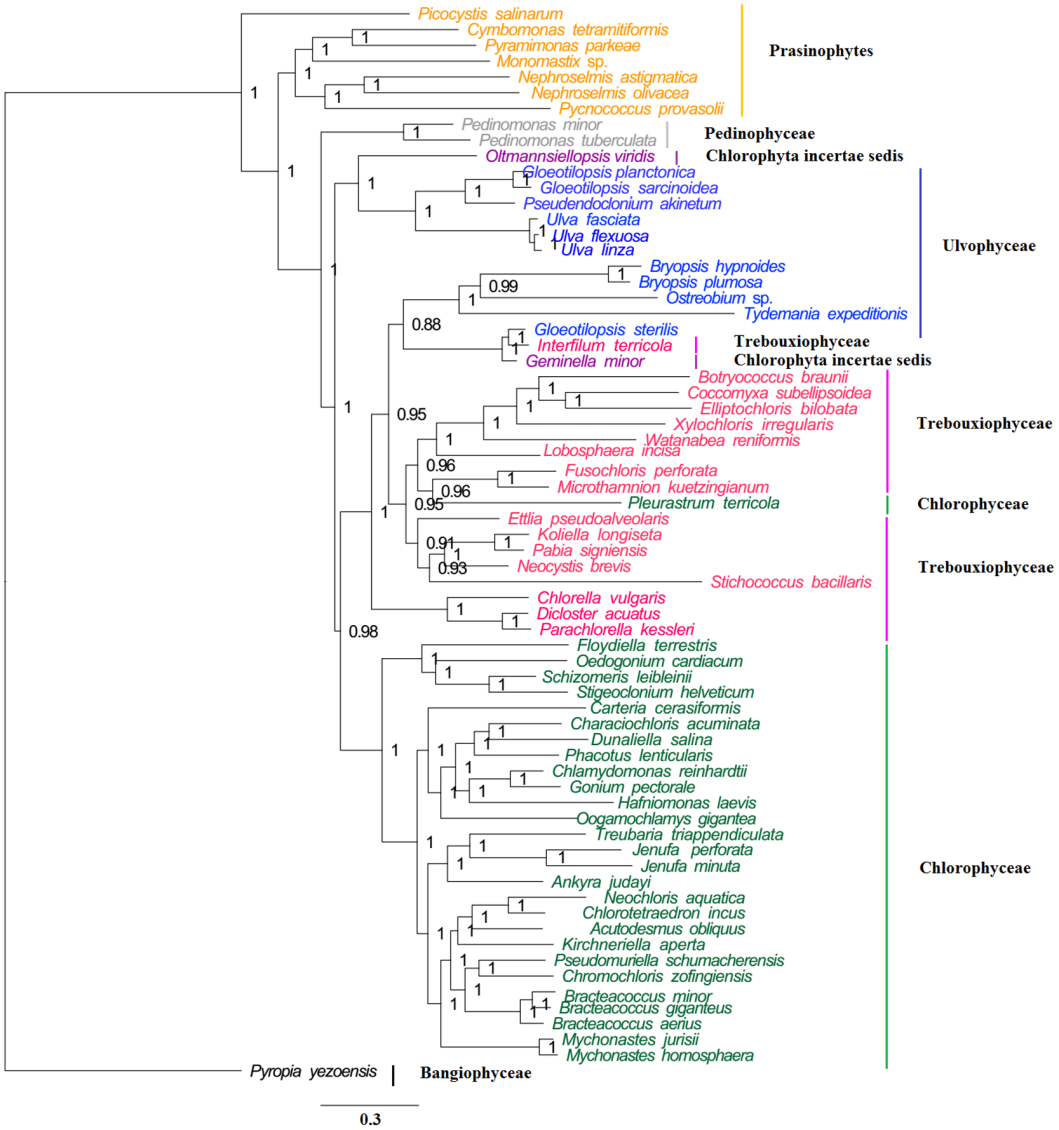
Bayesian 50% majority rule consensus tree based on 24 nucleotide sequences of 68 strains representing the Chlorophyta. The 24 genes were *atp*A, *atp*B, *atp*E, *atp*F, *pet*B, *pet*D, *pet*G, *psa*B, *psb*B, *psb*D, *psb*E, *psb*F, *psb*H, *psb*J, *psb*K, *psb*L, *psb*N, *rbc*L, *rpl*2, *rpl*20, *rpl*36, *rpo*A, *rps*8 and *ycf*3. The main parameters were: ngen = 2000000, samplefreq = 100, nchains = 4, nst = 6, rates = gamma. The Bayesian posterior probabilities were given at the nodes. The tree was rooted to *Pyropia yezoensis*.

To determine the phylogenetic position of *U*. *flexuosa* in Chlorophyta, 67 complete chloroplast genome sequences were obtained from the Genebank database, which belonged to Chlorophyceae (28), Trebouxiophyceae (17), Ulvophyceae (10), Prasinophytes (7), Pedinophyceae (2), Chlorophyta incertae sedis (2) and Bangiophyceae (1) respectively. The number shown in the parenthesis represents the number of species in the corresponding clade. To conduct phylogenetic analysis, we extracted 24 protein sequences, which were present among all the 68 chloroplast genomes. After alignment there were a total of 21534 positions in the final dataset. The phylogenetic tree showed that *U*. *flexuosa* and *U*. *linza* gathered into a cluster (Fig 5, S3 Fig).

### Rearrangements in the Chlorophyte cpDNA

**Fig 6:**
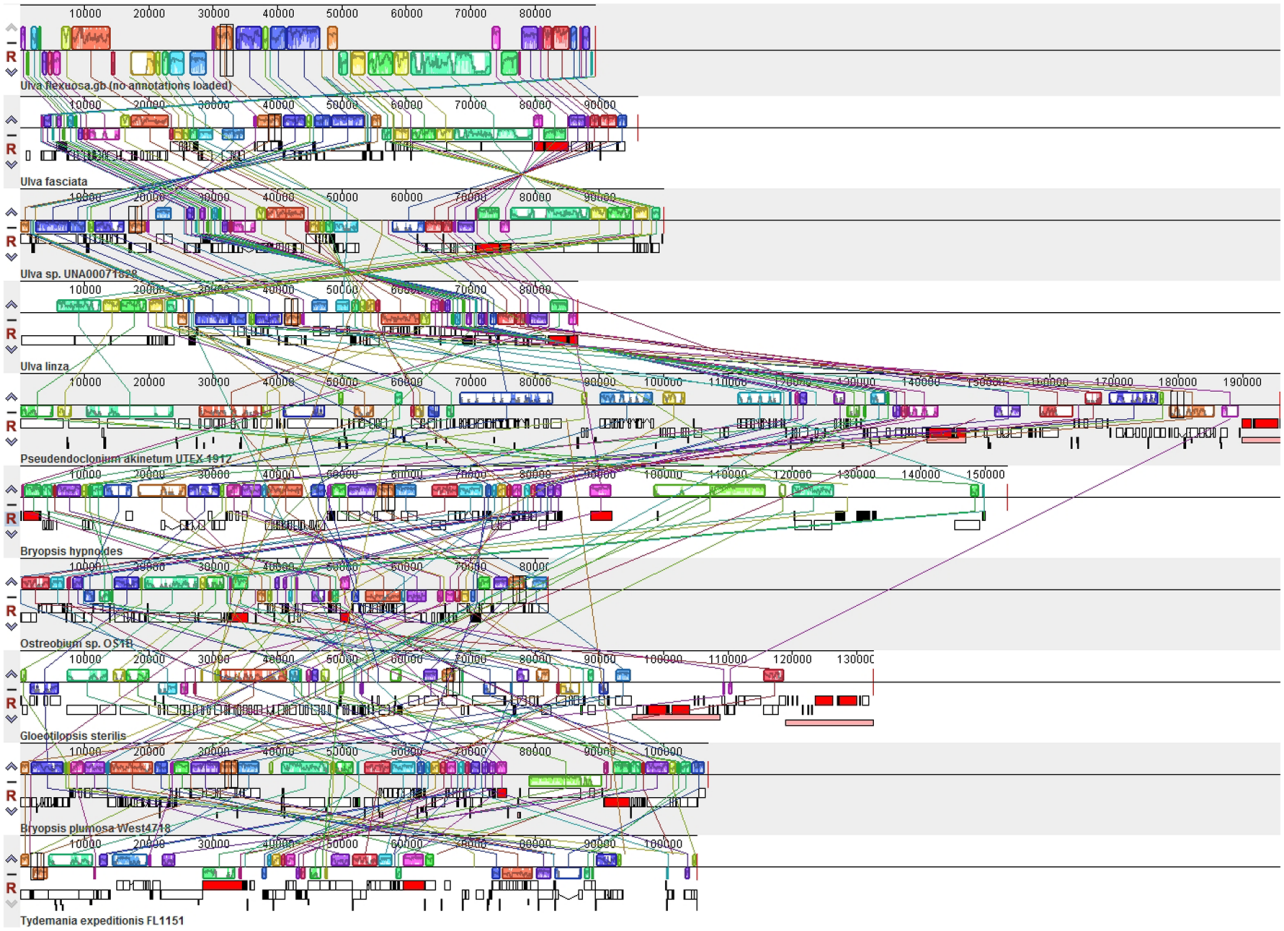
Mauve alignments of *U*. *flexuosa* cpDNA (top of alignment) with those of 9 chlorophytes.

Genome rearrangement analysis for the ten cpDNAs Mauve alignment from Ulvophyceae showed there was no significant difference in genomes rearrangement within *Ulva* spp. (Fig 6). However, the organellar genomes out of *Ulva* spp. across Ulvophyceae were highly rearranged comparing to *U*. *flexuosa*.

### Genome synteny analysis

**Fig 7:**
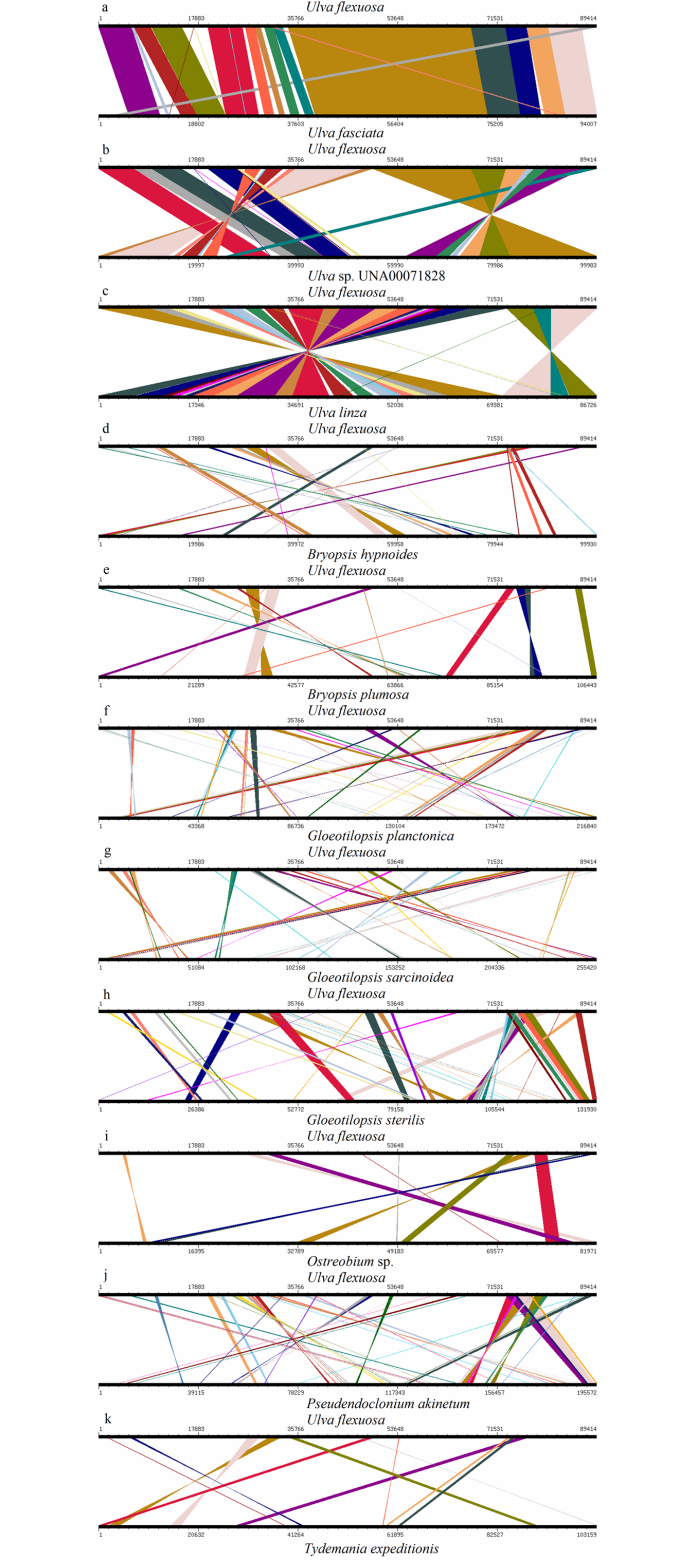
Comparative genomic analyses of 12 chloroplast genomes. The chloroplast genome of *U*. *flexuosa* was aligned with those of 12 species. Each horizontal black line represents a genome. The species names are shown next to the corresponding line. The conserved regions are bridged by lines.

Genome synteny was identified between the chloroplast genomes of *U*. *flexuosa* and 11 other species. The 11 species were classified into two groups based on degree of genome sequence similarity relative to the *U*. *flexuosa* chloroplast genome. The first group included *U*. *fasciata*, *Ulva* sp. UNA00071828 and *U*. *linza*. The chloroplast genome of this group was high conserved compared with that of *U*. *flexuosa* (Fig 7a–7c), with similarity above 91%. The second group included *B*. *hypnoides*, *B*. *plumosa*, *G*. *planctonica*, *G*. *sarcinoidea*, *G*. *sterilis*, *Ostreobium* sp., *P*. *akinetum* and *T*. *expeditionis* with similarity below 30% relative to *U*. *flexuosa* (Fig 7d–7k). These results suggested that genomes had high conservation among *Ulva* spp.

## Discussion

It indicated that the main genes of chloroplast genomes had high conservation among *Ulva* spp. which was monophyletic clade in Fig 5, with bayesian posterior probabilities (PP) of 1.0 and RAxML bootstrap support values (ML) of 100. These genomes might function similarly in photosynthesis, which were in line with their simultaneous originating, floating and outbreaking as green tide in the Yellow Sea of China. Meanwhile, the SSRs among *U*. *flexuosa* was also similar to those observed in three reported chloroplast genomes of other species belonging to Ulvaceae (Table 3), which provided potential molecular markers for *Ulva* spp. identification. Furthermore, there were no significant difference in genomes rearrangement (Fig 6) and genome synteny within cpDNAs from *Ulva* spp. (Fig 7). In a word, the *Ulva* spp. are conserve and usually monophyletic in phylogenetic tree [5].

**Fig 8:**
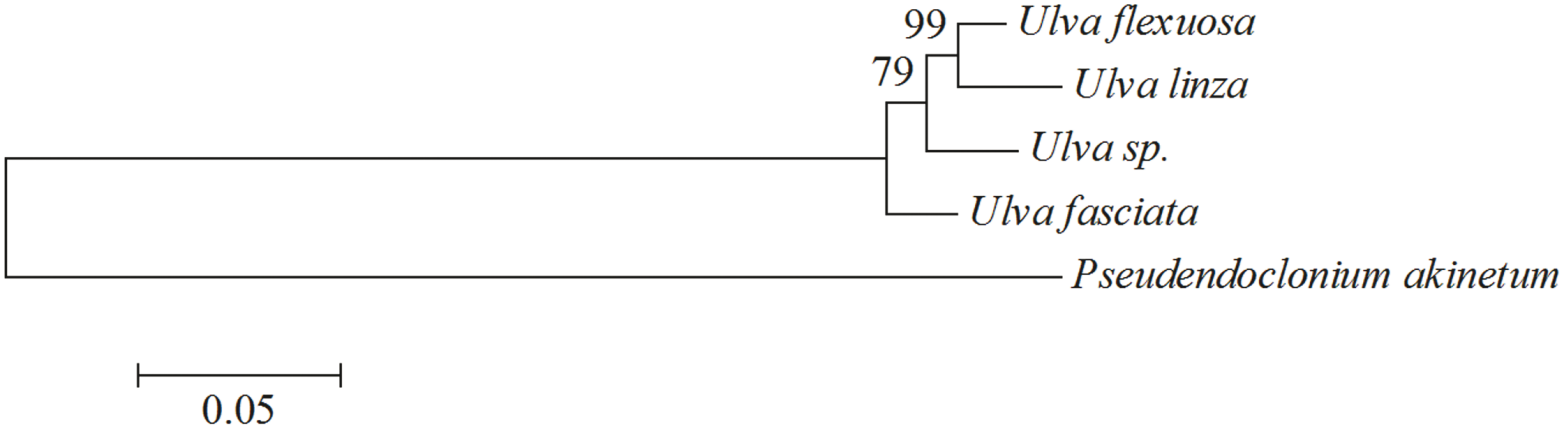
Molecular phylogenetic analysis of *Ulva* genus. The tree was constructed with the sequences of 67 coding genes present in all five species (*U*. *flexuosa*, *U*. *fasciata*, *U*. *linza*, *Ulva* sp. and *Monomastix* sp.) using the Maximum Likelihood method. *Monomastix* sp. was used as outgroup. Bootstrap supports were calculated from 1000 replicates.

Correlational studies [5, 33] within *Ulva* spp. are rarely seen, since few related genomes are reported to illustrate the evolutionary relationship [5, 13, 34, 35]. *U*. *flexuosa* is a cosmopolitan green algae encountered in fresh waters to saline waters [2], which may take on the characteristics of transition state and be supposed as a coordinate points for evolution [5]. In order to compare *U*. *flexuosa* with reported *Ulva* spp. in terms of chloroplast genome, we then made another phylogenetic tree for evolutionary analysis, including four kinds of species from *Ulva* spp. (*U*. *flexuosa*, *U*. *fasciata*, *U*. *linza* and *Ulva* sp. UNA00071828) and one exogenous species (*Monomastix* sp.) from prasinophytes. Since Ulvophyceae, Trebouxiophyceae, and Chlorophyceae shared a common ancestor which may have been prasinophyte-like [5]. 67 protein sequences (S6 Table) shared among all these five species were aligned using the ClustalW2 program. It was indicated that *U*. *flexuosa* was clustered with *U*. *linza* (Fig 8) in the phylogenetic tree, standing for the maximum homology between them. This was consistent with the phylogenetic tree in Fig 5 that *U*. *flexuosa* was clustered with *U*. *linza* (PP of 1.0 and ML of 100).

The close relationships between *U*. *flexuosa* and *U*. *linza* are supported by morphological and life history similarities to a certain degree. For example, the bright green thallus of two algae are tubular at the base but flattened above, in which cells are 11–15 μm in diameter. Besides, they are both alternate generations. However, we can still distinguish them easily according to the difference in morphology, since *U*. *linza* is linear or lanceolate banding, with wavy folds in the margin, while it is not the case in *U*. *flexuosa* (Fig 1). Thus nuclear genome may be the main factor which shapes the algae.

Molecular markers, including SSR, tandem repeats and forward repeats, are important supplement of morphology for species identification. These repeats were mainly distributed among intergenic spacers (S5 Table, Tables 2 and 4), which were variate more easily than coding sequences. The vast majority of repetitive sequence was composed of A/T, which was consistent with high A/T content (75%) in the genome. Therefore, whether high A/T content is related to the high variation is worthy studying.

**Fig 9:**
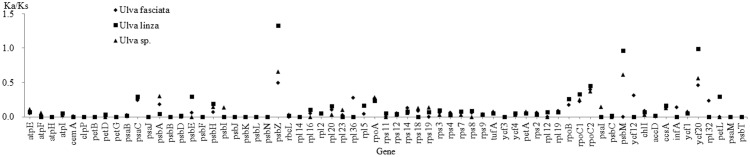
Comparison of selection forces (Ka/Ks) of the 67 common protein-coding genes in three-species matrix.

In order to find differential gene in *U*. *flexuosa* apart from other species among *Ulva* spp., a selection force and substitution rate assessment by 67 protein-coding genes (S6 Table) extracted from four species (*U*. *flexuosa*, *U*. *fasciata*, *U*. *linza* and *Ulva* sp. UNA00071828) using the KaKs_Calculator Toolbox 2.0 (https://sourceforge.net/projects/kakscalculator2) were performed using gamma-series methods of γ-NG and standard genetic code [36]. Taken *U*. *flexuosa* as control, most of Ka/Ks values were below one, which meant purify selection and conservative, wherein those of some genes (*atp*1, *atp*6, *cob*, *cox*2, *cox*3, *nad*6, *nad*7, *rpl*5, *rps*3 and *rps*10) in *U*. *linza* were zero. However, Ka/Ks values of *psb*Z from *U*. *linza* were 1.3, meaning positive selection, and those of *psb*M and *ycf*20 were near 1.0, meaning neutral evolution (Fig 9).

As gene of high variability, *psb*Z, *psb*M and *ycf*20 are potential molecular markers to identify *U*. *flexuosa* from *Ulva* spp. Besides, *psb*Z and *psb*M both belong to the subunits of photosystem II, involved in photosynthesis of photon absorption and energy transfer (Table 1). In previous research, we found that light and temperature were important factors which affected growth rate of green tide algae. For example, as high temperature species, the daily growth rate of *U*. *flexuosa* (including seedlings and adults) were higher than *U*. *prolifera*, *U*. *compressa* and *U*. *linza* under 25–35°C, or below 70 μmol·m^-2^·s^-1^ of light intensity [3]. Whether they are regulated by *psb*Z and *psb*M may worth studying in the next step.

## Conclusions

In the present study, we have: (1) sequenced the chloroplast genome of *U*. *flexuosa*; (2) annotated the chloroplast genome including ORFs and genetic code; (3) identified SSR, tandem repeats and forward repeats of the genome; (4) carried out a phylogenetic analysis of the 68 chloroplast genomes based on 24 conserved genes; (5) compared the chloroplast genome rearrangement of 10 species; (6) analyzed the synteny of *U*. *flexuosa* and 12 other species in Chlorophyte; and finally (7) comprehensively compared the chloroplast genome homology of four *Ulva* species. We found that organellar genomes in other species across Ulvophyceae were high rearranged comparing with *U*. *flexuosa*, while those among *Ulva* spp. were high conserved. Though *U*. *flexuosa* was closer to *U*. *linza* than *U*. *fasciata* and *Ulva* sp. UNA00071828 in phylogenetic tree, the average Ka/Ks between *U*. *flexuosa* and *U*. *linza* assessed by 67 protein-coding genes was higher than those between *U*. *flexuosa* and other species in *Ulva* genus, due to the variation of *psb*Z, *psb*M and *ycf*20. Future studies may include range of light, temperature and salinity on photosynthesis of *U*. *flexuosa* based on chloroplast genomes and transcriptome, as well as molecular identification of *Ulva* spp.

## Supporting information

S1 Table Primer sequence designed based on *Ulva linza* (NC_030312) as template for *U*. *flexuosa* cpDNAs sequencing. (DOCX)

S2 Table Adjustment primer sequence used for *U*. *flexuosa* cpDNAs sequencing. (DOCX)

S3 Table GenBank accession numbers used in the *rbc*L and *tuf*A phylogenetic trees. (DOCX)

S4 Table Codon usage and codon—anticodon recognition patterns in *U*. *flexuosa* chloroplast genome. (DOCX)

S5 Table Numbers of mono- and dinucleotide SSRs identified in the chloroplast genome of *U*. *flexuosa*. (DOCX)

S6 Table Names of 67 genes for comprehensively comparing the chloroplast genome homology of four *Ulva* species. (DOCX)

S1 Fig Genetic distances of the *rbc*L sequences of *Ulva compressa*, *Monostroma grevillei* (GU183089) as outgroups. (TIF)

S2 Fig Genetic distances of the *tuf*A sequences of *Ulva compressa*, *Monostroma grevillei* (HQ610262) as outgroups. (TIF)

S3 Fig Maximum Likelihood tree based on 24 nucleotide sequences of 68 strains representing the Chlorophyta. The 24 genes were *atp*A, *atp*B, *atp*E, *atp*F, *pet*B, *pet*D, *pet*G, *psa*B, *psb*B, *psb*D, *psb*E, *psb*F, *psb*H, *psb*J, *psb*K, *psb*L, *psb*N, *rbc*L, *rpl*2, *rpl*20, *rpl*36, *rpo*A, *rps*8 and *ycf*3. The appropriate model of evolution for each gene determined by jModelTest 2.1 was GTR+I+G, which was reassessed by PAUP 4.0a152 (P value = 1 − (99/100) = 0.010000). Then, the evolutionary history was inferred using the Maximum Likelihood method implemented in RaxML. The detailed parameters were “raxmlHPC-PTHREADS -f a -x 12345 -# 1000 -m GTRGAMMA -s dna.phy -n nex -p 12345 -q part.txt -T 6”. The tree with the final ML Optimization Likelihood (-548221.173374) was shown. The significance level for the phylogenetic tree was assessed by bootstrap testing with 1000 replications. The RAxML bootstrap support values were given at the nodes. The tree was rooted to Pyropia yezoensis.
(TIF)
