# Supplementary material for: Complete chloroplast genome of green tide algae Ulva flexuosa (Ulvophyceae, Chlorophyta) with comparative analysis
Source: PLoS One. 2017 Sep 1;12(9):e0184196. doi: 10.1371/journal.pone.0184196 (PMC5581003; doi:10.1371/journal.pone.0184196)
Supplement: S1 Table — (DOCX) [file pone.0184196.s001.docx]

**S1 Table Primer sequence designed based on *Ulva* sp. UNA00071828 as template for *Ulva flexuosa* cpDNAs sequencing**

| Primer | sequence | length | Primer | sequence | length |
| --- | --- | --- | --- | --- | --- |
| psbZ-1F | GTTTCATTATCTTTTATTCTTGTAATTGGTG | 31 | petA-1R | ATTGTCCACGACCTCTATTACCCC | 24 |
| petA-1F | CCTCAAGCAGTATTACCTGATAGTGTTTT | 29 | atpA-1R | GCTGAACCTACACGAGAAACAGAAATA | 27 |
| atpA-1F | TTAGAGGAACGAGGTTGTATGGATTAT | 27 | psbD-1R | GTAATAGTAATAAACCTGACCAACCAACA | 29 |
| psbD-1F | CTATCGCAATCGGTAAGTCAGAAGA | 25 | psbB-1F | CACGGAATAGTTCTAAATCCCACATAA | 27 |
| psbB-1R | TGACTCGTTTAGGTGTTACCCAATCT | 26 | ycf3-1F | TTTACCAACATCAAATAGAGAAAAACAA | 28 |
| rpl16-1F | TTTGCTACTGGTGTTGTTACTCCTCTA | 27 | ycf3-1R | TATCATACGGGTCTATTTCCAATCTTA | 27 |
| petB-1F | CCTACTTGGTCCCAGGGTAATGA | 23 | rpl16-1R | TTCCCGATAAACCTGTAACAATGC | 24 |
| psbE-1F | AAAAATGGCAGGGACTACAGGAGA | 24 | petB-1R | TTTATTATCGTCCAACAGTAGCAGAAG | 27 |
| psbA-1F | TGGGTAGAAGTGTAAACCAATAGCG | 25 | psaA-1R | TAAATGGAGATGTAGGAGGCGGTT | 24 |
| psaA-1F | GAAAAACATTGCTGCTGCCATTAC | 24 | rpoC2-1R | ATTGAACAACTTTTAGAAGCAAGAGAA | 27 |
| rpoC2-1F | CTTGAGATTGATAAACTTTTTGAACAGAA | 29 | rpoC2-2R | AATCGTTTAGTTTCTTTAGGTGAATCTGT | 29 |
| rpoC2-2F | CTTTGTCCTGAAAAAACACCACCA | 24 | rpoB-1R | GCTCGTTCAACTGGTCCTTATTCT | 24 |
| chll-2F | CCTTCACGCTCAACTGTATTCCAC | 24 | psbZ-1R | TAAAGCAAAAACTAATAAAACCCAAAT | 27 |
| CP14-1F | TAAATTATCGTAAATACCAAAAAATG | 26 | YGCP15-NR | CTTGTAAGGCAACCACTCTACCAAC | 25 |
| psbE-1R | TGTAATACTGTGAATAACCCAATAACGA | 28 | psbA-1R | TTTCAGTATTCATCATCGCATTTGTT | 26 |
| QCP13-1F | AAAAACTACCTAGAAATAGTTCTGCT | 26 | QCP13-2F | AGTAAATTGATGAACAATATGAAATA | 26 |
| QCP13-3F | ACAAGATGAGTTTTTTAATAAATCA | 25 | QCP13-4F | GTTTTGTAATAAAATTACCATTTGGAAT | 28 |
| QCP13-5F | ATCATAATAGTTTGATTCTCTTTTCG | 26 | QCP14-1F | AGTCTGAAATCTAATTATAGAAAAGCA | 27 |
| QCP14-2F | TAATTTTCAAGAATCCCCTTTT | 22 |  |  |  |
