# Supplementary material for: Complete chloroplast genome of green tide algae Ulva flexuosa (Ulvophyceae, Chlorophyta) with comparative analysis
Source: PLoS One. 2017 Sep 1;12(9):e0184196. doi: 10.1371/journal.pone.0184196 (PMC5581003; doi:10.1371/journal.pone.0184196)
Supplement: S2 Table — (DOCX) [file pone.0184196.s002.docx]

**S2 Table Adjustment primer sequence used for** *Ulva flexuosa* **cpDNAs sequencing**

| label | primer | expected length | handling |
| --- | --- | --- | --- |
| QCP2 | psbZ-1F/petA-1R | 6894 | breaking |
| QCP3 | petA-1F/atpA-1R | 3494 | breaking |
| QCP4 | atpA-1F/psbD-1R | 6036 | breaking |
| QCP5 | psbD-1F/psbB-1F | 1927 | breaking |
| QCP9 | ycf3-1F/psbB-1R | 3500 | breaking |
| QCP8 | rpl16-1F/ycf3-1R | 6397 | breaking |
| QCP7 | petB-1F/rpl16-1R | 7394 | breaking |
| QCP6 | psbE-1F/petB-1R | 6338 | breaking |
| QCP5B | psbE-1R/psbA-1R | 4000 | breaking |
| QCP11 | psbA-1F/psaA-1R | 7226 | breaking |
| QCP12 | psaA-1F/rpoC2-1R | 7202 | breaking |
| QCP13 | rpoC2-1F/rpoC2-2R | 6706 | breaking |
| QCP14 | rpoC2-2F/rpoB-1R | 6892 | breaking |
| QCP17C | chil-2F/psbZ-1R | 4000 | sequencing |
| QCP15N | CP14-1F/YGCP15-NR | 8000 | breaking |
