# Supplementary material for: Complete chloroplast genome of green tide algae Ulva flexuosa (Ulvophyceae, Chlorophyta) with comparative analysis
Source: PLoS One. 2017 Sep 1;12(9):e0184196. doi: 10.1371/journal.pone.0184196 (PMC5581003; doi:10.1371/journal.pone.0184196)
Supplement: S3 Table — (DOCX) [file pone.0184196.s003.docx]

**S3 Table GenBank accession numbers used in the *rbc*L and *tuf*A phylogenetic trees.**

| **Taxon** | ***rbc*L** | ***tuf*A** | **Taxon** | ***rbc*L** | ***tuf*A** |
| --- | --- | --- | --- | --- | --- |
| *Ulva arasakii* | AY004762 | AB561079 | *Ulva lobata* | HQ603604 | KM255006 |
| *Ulva armoricana* | AB097630 |  | *Ulva meridionalis* | AB598813 |  |
| *Ulva australis* | KC411886 | KC411859 | *Ulva ohiohilulu* |  | KT932977 |
| *Ulva beytensis* |  | KC661441 | *Ulva ohnoi* | KP279696 |  |
| *Ulva californica* | KP233750 | HQ610279 | *Ulva ovata* |  | KC661429 |
| *Ulva clathrata* | AY255862 |  | *Ulva pertusa* | AB894324 |  |
| *Ulva clathratioides* | EU933940 |  | *Ulva procera* | EF109991 | KM254997 |
| *Ulva compressa* | AF387106 | JN029296 | *Ulva prolifera* | AB598810 | HQ610403 |
| *Ulva erecta* |  | KC661427 | *Ulva reticulata* | HQ026496 | KC661444 |
| *Ulva fasciata* | EU933962 | JN029306 | *Ulva rigida* | EU484395 | HE600182 |
| *Ulva flexuosa* | EF110051 | JN029309 | *Ulva rotundata* | EU484401 |  |
| *Ulva gigantea* | EU484414 | HQ610297 | *Ulva scandinavica* | AY255870 |  |
| *Ulva howensis* | JN082214 |  | *Ulva stenophylla* |  | HQ610433 |
| [*Ulva iliohaha*](http://www.ncbi.nlm.nih.gov/nuccore/KT932976.1) |  | KT932976 | *Ulva stenophylloides* | EU933949 |  |
| *Ulva intestinalis* | EU933937 | JN029320 | *Ulva taeniata* | AY255875 | KC661451 |
| *Ulva lactuca* | AY422546 | HQ610326 | *Ulva tanneri* |  | KM255002 |
| *Ulva laetevirens* | EU933943 | JQ048942 | *Ulva torta* | AB830519 | HQ610437 |
| *Ulva linza* | AB741533 | EF595300 | *Blidingia minima* | AF387109 | HQ610329 |
| *Ulva flexuosa* | KX579943(To Be Provide) | | *Monostroma grevillei* | GU183089 | HQ610262 |
