# Supplementary material for: Complete chloroplast genome of green tide algae Ulva flexuosa (Ulvophyceae, Chlorophyta) with comparative analysis
Source: PLoS One. 2017 Sep 1;12(9):e0184196. doi: 10.1371/journal.pone.0184196 (PMC5581003; doi:10.1371/journal.pone.0184196)
Supplement: S4 Table — (DOCX) [file pone.0184196.s004.docx]

S4 Table Codon usage and codon–anticodon recognition patterns in *U. flexuosa* chloroplast genome

| Codon | Amino acid | Number | tRNA | Codon | Amino acid | Number | tRNA | Codon | Amino acid | Number | tRNA |
| --- | --- | --- | --- | --- | --- | --- | --- | --- | --- | --- | --- |
| GCA | A | 408 | *trnA-UGC* | AAG | K | 168 |  | AGC | S | 47 | *trnS-GCU* |
| GCC | A | 38 |  | CTA | L | 128 | *trnL-UAG* | AGT | S | 347 |  |
| GCG | A | 41 |  | CTC | L | 28 |  | TCA | S | 528 | *trnS-UGA* |
| GCT | A | 534 |  | CTG | L | 24 |  | TCC | S | 28 | *trnS-GGA* |
| TGC | C | 25 | *trnC-GCA* | CTT | L | 191 |  | TCG | S | 50 |  |
| TGT | C | 165 | *trnC-ACA* | TTA | L | 1932 | *trnL-UAA* | TCT | S | 415 |  |
| GAC | D | 90 | *trnD-GUC* | TTG | L | 97 | *trnL-CAA* | ACA | T | 514 | *trnT-UGU* |
| GAT | D | 638 |  | ATG | M | 365 | *trnM-CAU* | ACC | T | 41 | *trnT-GGU* |
| GAA | E | 833 | *trnE-UUC* | AAC | N | 237 | *trnN-GUU* | ACG | T | 60 | *trnT-CGU* |
| GAG | E | 84 |  | AAT | N | 1614 |  | ACT | T | 396 |  |
| TTC | F | 190 | *trnF-GAA* | CCA | P | 331 |  | GTA | V | 383 |  |
| TTT | F | 1218 |  | CCC | P | 39 |  | GTC | V | 21 | *trnV-GAC* |
| GGA | G | 322 |  | CCG | P | 45 |  | GTG | V | 32 |  |
| GGC | G | 60 | *trnG-GCC* | CCT | P | 311 | *trnP-UGG* | GTT | V | 610 |  |
| GGG | G | 80 |  | CAA | Q | 637 | *trnQ-UUG* | TGG | W | 282 | *trnW-CCA* |
| GGT | G | 763 |  | CAG | Q | 63 |  | TAC | Y | 114 | *trnY-GUA* |
| CAC | H | 83 | *trnH-GUG* | AGA | R | 239 | *trnR-UCU* | TAT | Y | 821 |  |
| CAT | H | 265 |  | AGG | R | 34 |  | TAA | * | 116 |  |
| ATA | I | 522 |  | CGA | R | 103 |  | TAG | * | 35 |  |
| ATC | I | 119 | *trnI-CAU*  *trnfM-CAU* | CGC | R | 35 |  | TGA | * | 21 |  |
| ATT | I | 1267 |  | CGG | R | 11 |  |  |  |  |  |
| AAA | K | 1806 | trnK-UUU | CGT | R | 411 | *trnR-ACG* |  |  |  |  |
