# Supplementary material for: Complete chloroplast genome of green tide algae Ulva flexuosa (Ulvophyceae, Chlorophyta) with comparative analysis
Source: PLoS One. 2017 Sep 1;12(9):e0184196. doi: 10.1371/journal.pone.0184196 (PMC5581003; doi:10.1371/journal.pone.0184196)
Supplement: S5 Table — (DOCX) [file pone.0184196.s005.docx]

S5 Table Numbers of mono- and dinucleotide SSRs identified in the chloroplast genome of ***U.*** *flexuosa*

| Repeat  Types | Number of Copies | | | | | | | | | | | |
| --- | --- | --- | --- | --- | --- | --- | --- | --- | --- | --- | --- | --- |
|  | 4 | 5 | 6 | 7 | 8 | 9 | 10 | 11 | 12 | 13 | 18 | Total |
| A/T |  |  |  |  | 84 | 49 | 19 | 8 | 5 | 1 | 1 | 167 |
| AG/CT | 3 |  |  |  |  |  |  |  |  |  |  | 3 |
| AT/AT | 20 | 10 | 5 | 1 | 1 |  |  |  |  |  |  | 37 |
