# Supplementary material for: Complete chloroplast genome of green tide algae Ulva flexuosa (Ulvophyceae, Chlorophyta) with comparative analysis
Source: PLoS One. 2017 Sep 1;12(9):e0184196. doi: 10.1371/journal.pone.0184196 (PMC5581003; doi:10.1371/journal.pone.0184196)
Supplement: S6 Table — (DOCX) [file pone.0184196.s006.docx]

S6 Table Names of 67 genes for comprehensively comparing the chloroplast genome homology of four *Ulva species*

atpE, atpF, atpH, atpI, cemA, clpP, petB, petD, petG, psaB, psaC, psaJ, psbA, psbB, psbD, psbE, psbF, psbH, psbI, psbJ, psbK, psbL, psbN, psbZ, rbcL, rpl14, rpl16, rpl2, rpl20, rpl23, rpl36, rpl5, rpoA, rps11, rps12, rps14, rps18, rps19, rps3, rps4, rps7, rps8, rps9, tufA, ycf3, ycf4, petA, rps2, rpl12, rpl19, rpoB, rpoC1, rpoC2, psaI, psbC, psbM, ycf12, chlI, accD, ccsA, infA, ycf1, ycf20, rpl32, petL, psaM, psbT
