# Supplementary figures and images for: Complete chloroplast genome of green tide algae Ulva flexuosa (Ulvophyceae, Chlorophyta) with comparative analysis
Source: PLoS One. 2017 Sep 1;12(9):e0184196. doi: 10.1371/journal.pone.0184196 (PMC5581003; doi:10.1371/journal.pone.0184196)

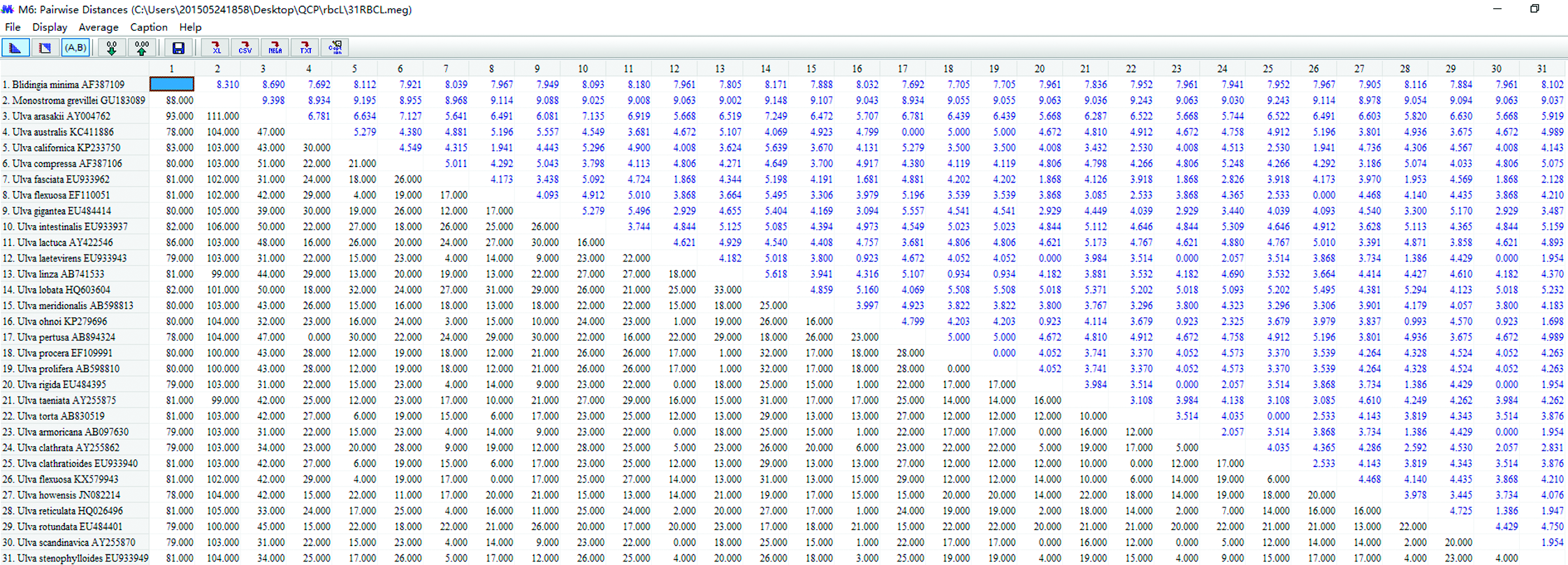

Supplement: S1 Fig — (TIF) [file pone.0184196.s007.tif]

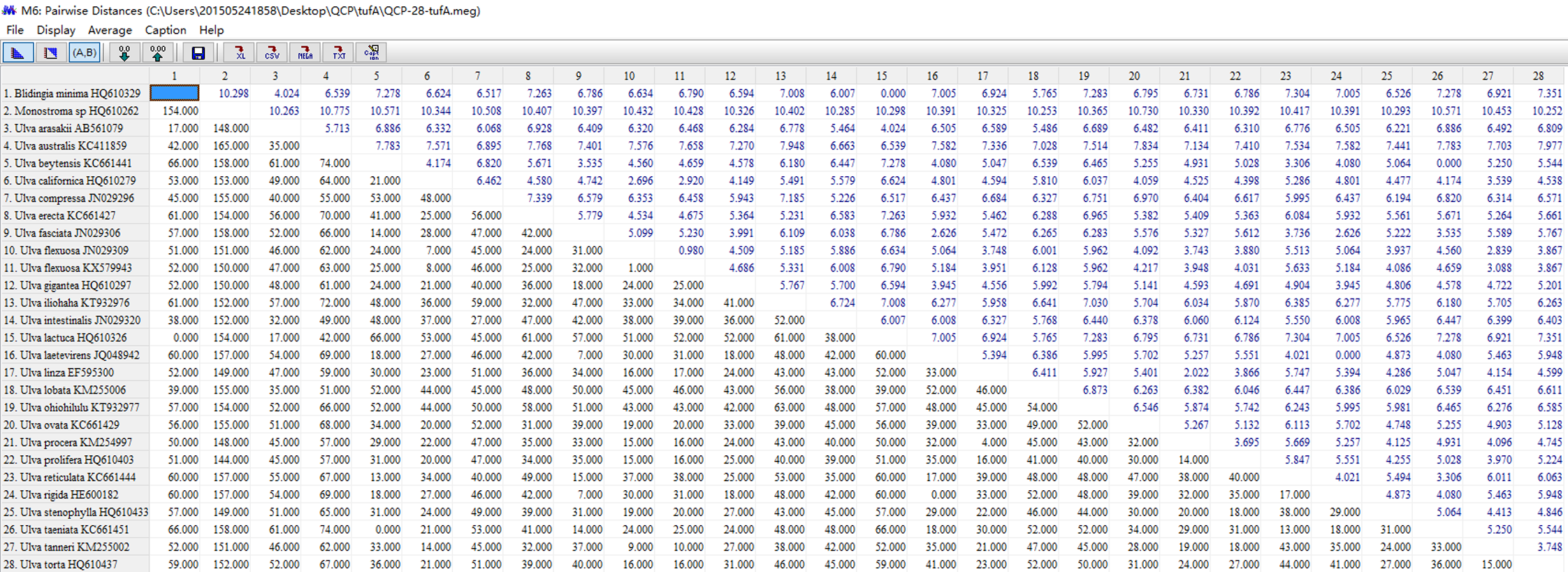

Supplement: S2 Fig — (TIF) [file pone.0184196.s008.tif]

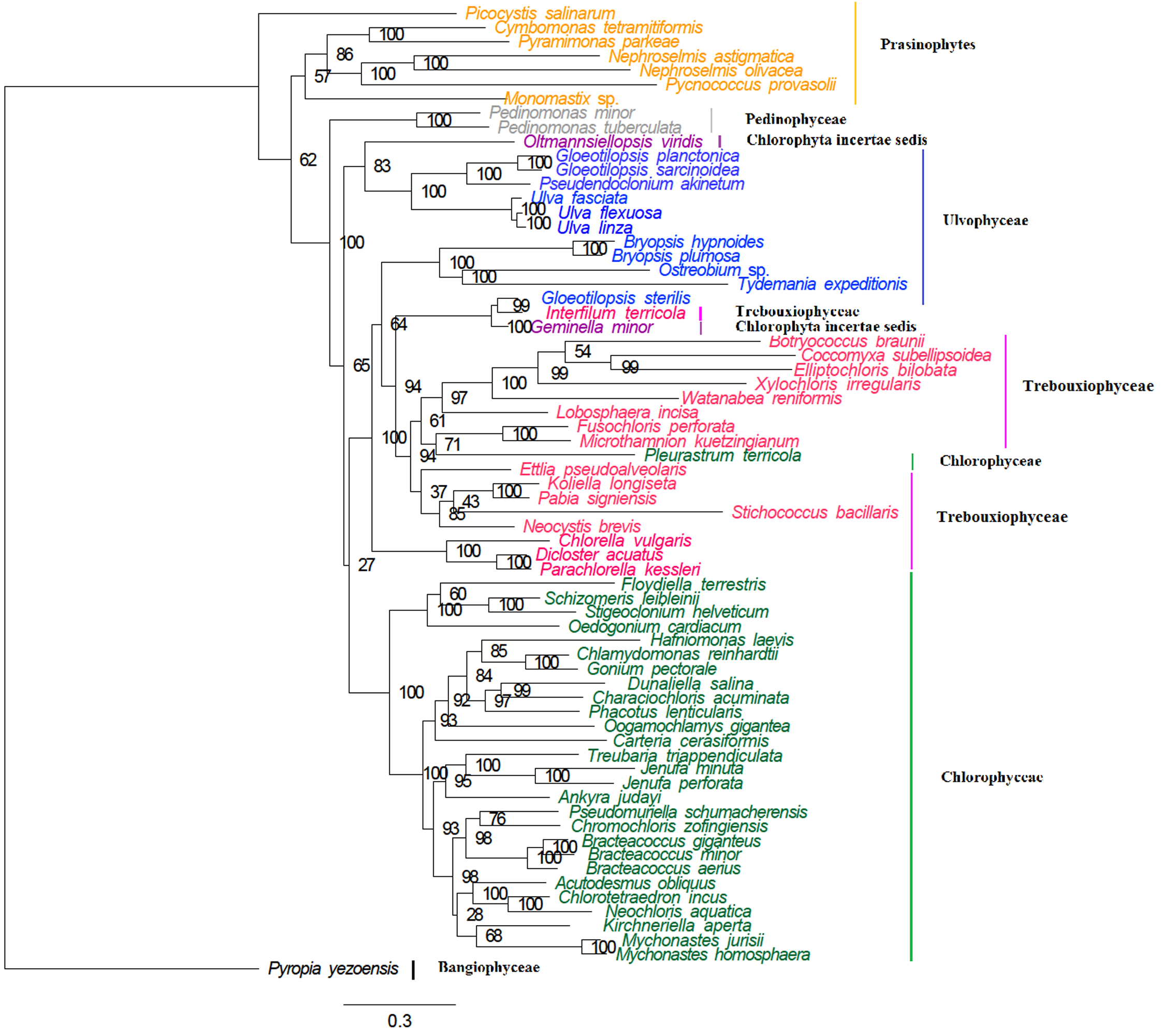

Supplement: S3 Fig — The 24 genes were atpA, atpB, atpE, atpF, petB, petD, petG, psaB, psbB, psbD, psbE, psbF, psbH, psbJ, psbK, psbL, psbN, rbcL, rpl2, rpl20, rpl36, rpoA, rps8 and ycf3. The appropriate model of evolution for each gene determined by jModelTest 2.1 was GTR+I+G, which was reassessed by PAUP 4.0a152 (P value = 1 − (99/100) = 0.010000). Then, the evolutionary history was inferred using the Maximum Likelihood method implemented in RaxML. The detailed parameters were “raxmlHPC-PTHREADS -f a -x 12345 -# 1000 -m GTRGAMMA -s dna.phy -n nex -p 12345 -q part.txt -T 6”. The tree with the final ML Optimization Likelihood (-548221.173374) was shown. The significance level for the phylogenetic tree was assessed by bootstrap testing with 1000 replications. The RAxML bootstrap support values were given at the nodes. The tree was rooted to Pyropia yezoensis. (TIF) [file pone.0184196.s009.tif]
